# Immunogenic Cell Death in Hematological Malignancy Therapy

**DOI:** 10.1002/advs.202207475

**Published:** 2023-02-23

**Authors:** Zhaoyun Liu, Xintong Xu, Kaining Liu, Jingtian Zhang, Dan Ding, Rong Fu

**Affiliations:** ^1^ Department of Hematology Tianjin Medical University General Hospital Tianjin 300052 P. R. China; ^2^ State Key Laboratory of Medicinal Chemical Biology Key Laboratory of Bioactive, Materials, Ministry of Education and College of Life Sciences Nankai University Tianjin 300071 P. R. China

**Keywords:** aggregation‐induced emission, immunogenic cell death, multiple myeloma, photodynamic therapy, reactive oxygen species

## Abstract

Although the curative effect of hematological malignancies has been improved in recent years, relapse or drug resistance of hematological malignancies will eventually recur. Furthermore, the microenvironment disorder is an important mechanism in the pathogenesis of hematological malignancies. Immunogenic cell death (ICD) is a unique mechanism of regulated cell death (RCD) that triggers an intact antigen‐specific adaptive immune response by firing a set of danger signals or damage‐associated molecular patterns (DAMPs), which is an immunotherapeutic modality with the potential for the treatment of hematological malignancies. This review summarizes the existing knowledge about the induction of ICD in hematological malignancies and the current research on combining ICD inducers with other treatment strategies for hematological malignancies.

## Graphical abstract

This article introduces the concept of immunogenic death and current therapy can lead to immunogenic death in hematologic malignancy disease therapy, which will open up new ideas for the diseases treatment.

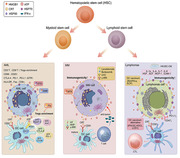

## 1 Introduction

Accidental cell death (ACD) and RCD are the main mechanisms of cell death. In contrast to the unpredictable and uncontrolled biological behavior of ACD, RCD has clear molecular mechanisms and signaling cascades. According to current knowledge, RCD can be broadly classified as including necrotic apoptosis, thermal apoptosis, iron apoptosis, and ICD, among other forms.^[^ ^1^ ^]^

Cells that through death, stress, or injury release molecules or expose them on their surface and can act as danger signals for the immune system are known as DAMPs.^[^ ^2^ ^]^ DAMPS can be released or secreted as ATP and HMGB1, and some DAMPs molecules, such as CRT and HSP70/90, are exposed or enriched on the surface of tumor cells.^[^ ^3^ ^]^ During the cell death phase, other DAMPs are produced as terminal breakdown products (e.g., uric acid) and are eventually degraded.^[^ ^4^, ^5^ ^]^ Finally, DAMP danger signals combine with pattern recognition receptors (PRRs) and are displayed on innate immune cells, such as natural killer (NK) cells and dendritic cells (DCs), which activate the adaptive immune system by promoting the maturation and activation of these cells.^[^ ^6^ ^]^ In addition to DAMPs, microbial‐associated molecular patterns (MAMPs), which consist of microbial nucleic acid species and structural components, can also mediate immunostimulatory effects mainly through PRRs in mammalian organisms.^[^ ^7^ ^]^

Currently, chemotherapy and immunotherapy are the two main treatment modalities for hematological malignancies.^[^ ^8^ ^]^ Management of hematological malignancies has traditionally relied on chemotherapy regimens; however, while chemotherapy can benefit some patients to some extent, it still does not lead to complete remission, and new problems such as relapse and drug resistance have arisen. Immunotherapeutic regimens based on the immune system of patients with hematological malignancies represent a therapeutic mode that has become a current research hotspot. ICD is an immunotherapeutic modality with potential for the treatment of hematological malignancies. Therefore, focusing on and developing the induction of ICD in hematological malignancies and exploring the therapeutic possibilities in hematological tumors is certainly a new treatment strategy of interest.

This paper reviews the existing models of ICD induction in hematological malignancies and the combination of ICD induction agents with other treatments, which provides new insights into the clinical treatment of hematological malignancies.

## 2 Immunogenic Cell Death

**Figure 1:**
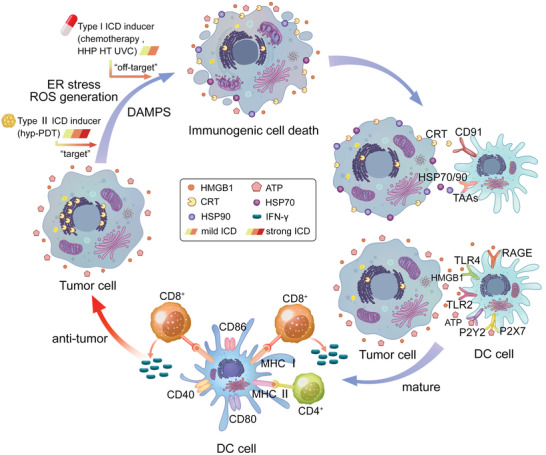
Immunogenic cell death. The key to the development of ICD is the coordinated emission of DAMPs, a process including translocation of ER chaperones on the cell surface (CRT, HSP70, and 90), secretion of ATP activity, and release of HMGB1 from the nucleus into the extracellular environment. After stimulation by ICD induction in tumor cells, CRT can be exposed to the outer lobe of the cytoplasmic membrane, and functions as an “eat me” signal; it binds to CD91 to activate the DC, then participates in antigen cross‐linking presentation. The released ATP can act as a “find me” signal; it is sensed by the P2Y2 and P2×7 receptors on APCs and induces recruitment to the apoptotic site, subsequently resulting in the secretion of mature IL‐1*β* and IL‐18. HMGB1 plays a powerful immunostimulatory role by interacting with several PRRs (TLR2, TLR4, and RAGE). HSP70 and HSP90 on the membrane surface can bind to TAAs, thus stimulating DC maturation, and activating both CD4 and CD8 T cells. Finally, the release of the type I interferons/IL‐1 family kills the tumor cells. ICD, immunogenic cell death; DAMPs, damage‐associated molecular patterns; ER, endoplasmic reticulum; CRT, calreticulin; HSP, heat shock protein; HMGB1, high mobility mass box 1; DC, dendritic cell; APC, antigen‐presenting cell; PRR, pattern recognition receptors; TAA, tumor‐associated antigen; IL‐1, interleukin‐1.

The concept of ICD was first proposed in 2005^[^ ^9^ ^]^ and was defined by the Cell Death Nomenclature Committee as “a form of RCD sufficient to activate an adaptive immune response in immune‐functional homologous hosts.” This definition clarifies that under certain specific circumstances, functionally unique stress can induce an inflammatory response in RCD and ultimately establish long‐term immune memory by stimulating an adaptive immune response driven by cytotoxic T cells. Immunogenicity ultimately determines the propensity of the dying cells to drive an immune response. Adjuvant properties arise from the coordinated release or exposure of danger signaling molecules that through continuous accumulation, eventually form DAMPs that are integral to the maturation and recruitment functions of antigen‐presenting cells (APCs), the strength of which is correlated with the strength of the dying cells and danger signaling molecules that trigger them.^[^ ^10^, ^11^, ^12^ ^]^ This means that adjuvants and antigenicity ultimately determine the ability of ICD cells to drive adaptive immunity in the microenvironment.^[^ ^5^ ^]^ In terms of the mechanism of ICD, although tumor cells all contain the organelles (ER and mitochondria) required for ICD induction, and many have sufficient DAMPs for APC stimulation, there is still significant variability in the intensity of intracellular responses generated by stressor induction, and not all responses are successful in driving ICD. During the generation of ICD, the molecules involved in ICD regulation have various specific roles (**Figure** **1**):

1. The CRT protein is a highly conserved, soluble, ER‐associated chaperone protein located predominantly on the outer surface of the cytoplasm and cell membrane that functions both inside and outside the ER.^[^ ^13^, ^14^ ^]^ CRT exposed on the outer leaflet of the plasma membrane under stress conditions (called ecto‐CRT) acts as a powerful “eat me” signal by binding to LRP1 (also known as CD91), activating DCs, and participating in the antigen cross‐presentation process. Simultaneously, under the effects of some chemotherapy drugs, CRT exposed on the surface of cancer cells undergoing ICD is engulfed by DCs, resulting in a tumor‐specific cytotoxic T lymphocyte (CTL) response.^[^ ^15^, ^16^ ^]^ The pathways of CRT exposure differ according to the apoptotic phase and inducing stimuli.^[^ ^12^ ^]^ The presence of ecto‐CRT exposure induced in the pre‐apoptotic phase (phosphatidylserine‐free externalization and plasma membrane permeabilization) depends on ER transport to the Golgi, proximal control by protein kinase‐like endoplasmic reticulum kinase (PERK), and the pi3k‐mediated distal secretion pathway. The general transport of ecto‐CRT from the ER and Golgi membrane to the cell surface begins during early apoptosis.^[^ ^17^ ^]^ In late apoptosis, HSP70/90, located on the membrane surface, can bind to tumor‐associated antigens (TAAs), thus stimulating DC maturation.^[^ ^18^ ^]^

2. In a normal physiological state, externally released ATP can be an important means of regulating different cellular functions. In apoptotic cells, the released ATP acts as a “find me” signal and is sensed by P2Y2 and P2×7 receptors on APCs, inducing their recruitment to apoptotic sites. ATP simultaneously activates CASP1‐dependent NLRP3 inflammasome formation and subsequently causes secretion of mature interleukin (IL)‐1*β* and IL‐18.^[^ ^19^, ^20^ ^]^ The combined effects of the apoptotic phase and the stimulus that induces ATP secretion to determine the transport mechanism. In the absence of plasma membrane permeability, and like ecto‐CRT, ATP secretion during the pre‐apoptotic phase depends only on classical pi3k‐dependent extracellular secretion and PERK‐regulated proximal secretion pathways, but not on Bax or BAK8. When induced by various chemotherapeutic agents, phosphatidylserine is exposed on the plasma membrane and tumor cells release extracellular ATP. In early apoptosis (without plasma membrane permeability), ATP secretion may be dependent on pannexin 1 channels or autophagy; however, in mid‐ to late‐stage apoptosis (with plasma membrane permeability), ATP is passively secreted due to plasma membrane defects.^[^ ^21^, ^22^, ^23^ ^]^

3. HMGB1 is a non‐histone chromatin‐binding protein primarily located in the nucleus that plays a role in regulating the transcriptional activity of proteins, promoting VD(J) recombination, and regulating transcription at the chromatin level.^[^ ^6^, ^7^, ^24^ ^]^ Intra‐ and extracellularly, HMGB1 performs different functions. Intracellular HMGB1 interacts with Beclin‐1 to mediate autophagy. Extracellular HMGB1 activates macrophages and monocytes under the influence of proinflammatory molecules, exerts cytokine‐based functions, and participates in ICD immunogenesis.^[^ ^18^, ^24^ ^]^ HMGB1 can be released from necrotic cells and act as a DAMP, interacting with different PRRs (such as Toll‐like receptor 2 [TLR2] on DCs or TLR4) and RAGE, then exert potent immunostimulatory effects.^[^ ^25^ ^]^ The release of the proinflammatory factor HMGB1 from the nucleus of the extracellular space induced by ICD mainly occurs in late apoptosis, with blocking of the Z‐VAD‐FMK pathway delaying secondary necrosis.^[^ ^26^, ^27^ ^]^ Previously, caspase‐dependent oxidation of high mobility groups was found to occur during HMGB1 apoptosis, suggesting that HMGB1 activity may be regulated through redox modifications and mitochondrial reactive oxygen species (ROS) production.^[^ ^4^, ^28^ ^]^ Finally, in the final stage of ICD in apoptotic cells, members of the type I interferon (IFNs)/IL‐1 family are released and participate in the immune activation process after release.

Therefore, the occurrence of ICD requires the synergy of various factors such as CRT, ATP, HMGB1, and HSP70/90, and the ICD reaction can occur at all stages of apoptosis. It should also be noted that, in addition to ATP, HMGB1, and CRT, other molecules such as Bcl‐2, cyclophilin A, F‐actin, hepatoma‐derived growth factor, and HMGN1 can also be involved in the induction of ICD and play an immunomodulatory role similar to that of DAMPs associated with various cell death types.^[^ ^12^ ^]^

## 3 Inducing ICD

In recent years, ICD inducers have been widely reported to reduce tumor load by enhancing the immunogenicity of tumor cells and exerting anti‐tumor immune responses. The coexistence of ER stress and ROS generation is important for ICD induction. The activation of ER stress is also known as the unfolded protein response (UPR), and the PERK‐mediated UPR arm is essential for most ICD processes. Under basal conditions, the UPR is inactive; however, under stress conditions, three membrane‐bound sensors (PERK, inositol‐requiring enzyme 1 [IRE1], and activating transcription factor 6 [ATF6]) play roles in initiating the UPR process.^[^ ^29^, ^30^, ^31^ ^]^ Many intrinsic factors (such as carcinogenic activation, genetic alteration, and deteriorating secretion capacity) and external factors (such as hypoxia, acidosis, and nutrient deprivation) can cause ER stress, which can activate UPR.^[^ ^32^ ^]^ Galluzzi et al.^[^ ^33^ ^]^ proposed in 2017 that a number of methods for inducing ICDs are known, and each type of inducing stimulus is associated with the emission of its corresponding danger signal, including the following methods:

(1) Pathogens: Currently known relevant stressors include specialized intracellular antigens (e.g., multiple cellular and viral species), therapeutic lysozyme viruses, and related molecules with lysis potential (e.g., LTX‐315 and LTX‐401).^[^ ^34^, ^35^ ^]^ Experimental in vivo and in vitro studies related to intestinal inflammation (e.g., of *Salmonella enterica*, *Escherichia coli*, etc.) have revealed that antigen presentation by bone marrow‐derived DCs (BMDC) can be specifically regulated by Toll‐like receptor (TLR) signaling from pathogen‐containing phagosomes and facilitate microbial antigen presentation.^[^ ^35^ ^]^ After pathogen invasion, danger signals are transmitted intracellularly and in the microenvironment through autophagy and activation of the UPR, which stimulates the PRR to drive the secretion of proinflammatory cytokines.^[^ ^36^, ^37^ ^]^ A variety of microbial components, such as lipopolysaccharides, lipophilin, and flagellin, can be rapidly detected by dedicated TLRs, cytoplasmic DNA sensors, RIG‐I‐like receptors, or NOD‐like receptors.^[^ ^38^, ^39^ ^]^ In addition, in some specific cells (e.g., macrophages), the activation of inflammatory vesicles containing dying bacterial cells determines the ability of the macrophage to fight infection and ultimately promotes the secretion of cytokines.^[^ ^40^ ^]^ As a new therapeutic mode, measles virus in a randomized clinical trial enhanced innate antitumor activity on the one hand and exerted a specific adaptive immune response on the other by mediating ICD of melanoma cells.^[^ ^41^ ^]^ In addition, related studies found that the tumorolytic peptide LTX‐315 could play an immune‐dependent therapeutic role by mediating ICD through Bax/Bak‐regulated mitochondrial membrane permeabilization, and played a similar role to the anthraquinone drug mitoxantrone in inducing myeloid cell and T lymphocyte infiltration.^[^ ^42^, ^43^ ^]^ LTX‐401, a lysogenic amino acid derivative with immunogenic properties, was shown to be selectively enriched in the Golgi and to play a role upstream of mitochondrial membrane permeabilization to induce ICD.^[^ ^44^ ^]^ All major hallmarks of ICD, including CRT protein exposure, ATP release, HMGB1 efflux, and type I interferon production, were detectable during LTX‐315 and LTX‐401 ICD induction.

(2) Chemotherapy: As observed in mouse models, chemotherapy‐driven ICD‐dependent eIF2a phosphorylation of ER chaperones including CRT, ERp57, and HSP70/90 can expose the plasma membrane of dying cells. Chemotherapeutic agents can help the secretion and migration of cxc‐chemokine ligand 10 (CXCL10), HMGB1, and membrane‐associated protein A1 (ANXA1) and mediate autophagy‐related ATP secretion by activating IFN in tumor cells.^[^ ^45^, ^46^, ^47^ ^]^ Currently, anthracyclines such as doxorubicin, epirubicin, and idarubicin are the most potent antitumor agents for the induction of ICD in human tumor cells, as confirmed by studies in human prostate cancer, ovarian cancer, acute lymphoblastic leukemia cells, and mouse tumor cells.^[^ ^9^, ^46^ ^]^ Other common chemotherapeutic agents that can induce ICD are anthraquinones like mitoxantrone and hypericin, certain DNA damaging agents like cyclophosphamide and oxaliplatin,^[^ ^48^ ^]^ oxradiones, poly A‐ribose polymerase (PARP) inhibitors, mitotoxic agents such as docetaxel and patipilone, and peptides like bortezomib and carfilzomib.^[^ ^49^, ^50^ ^]^ In addition to conventional chemotherapeutic agents, several targeted anti‐tumor drugs and epigenetic modifiers as well as a variety of chemicals (e.g., the ubiquitin‐specific peptidase inhibitor Spautin‐1, the herbal ingredient shikonin, and the neurotoxin capsaicin) have been found to play an ICD‐inducing role in solid tumors such as malignant pleural mesothelioma and bladder cancer.^[^ ^33^, ^51^, ^52^ ^]^

(3) Physical cues activate ICD: irradiation, high hydrostatic pressure (HHP), near‐infrared photoimmunotherapy, severe toxic heat shock and hypericin‐based photodynamic therapy (PDT) are common physical inducers of ICD.^[^ ^53^, ^54^, ^55^ ^]^ Currently, known physical cue‐related ICD inducers are mainly used in experiments related to solid tumors.^[^ ^56^ ^]^ There are preclinical studies of hyperthermia (HT) for the treatment of melanoma,^[^ ^57^ ^]^ photothermal therapy (PTT) in neuroblastoma,^[^ ^58^ ^]^ an MB49 bladder cancer mouse model,^[^ ^59^ ^]^ PDT in subcutaneous and lung metastasis mouse models,^[^ ^60^ ^]^ and PDT and programmed death ligand 1 (PD‐L1) inhibitors in a colorectal cancer mouse model. HT induces apoptosis, activates the immune response, kills tumor cells, inhibits tumor growth, and prolongs the lifespan of mice. Thus, it has shown excellent preclinical efficacy. It was previously demonstrated in vaccination studies that *γ*‐irradiation and UVC light can kill tumor cells in mice and establish a protective immune response memory in immunocompetent hosts.^[^ ^55^ ^]^ *α*‐irradiation with 213Bi particles is now included in the list of ICD inducers, and induction of ICD by irradiation has been shown by vaccination experiments in mouse models of colorectal cancer to depend mainly on CRT exposure and ATP release, but the effect of other major markers of ICD on the ability to induce irradiation is not yet clear.^[^ ^61^, ^62^, ^63^ ^]^ Radiotherapy and chemotherapy generate corresponding immune responses by driving ICD responses and CD8+ T cell activation, whereas PDT and HHP alone upregulate various DC activation markers.^[^ ^12^, ^64^, ^65^ ^]^

(4) Necroptosis: It is not only a form of cell death but also triggers an antigen‐specific immune response.^[^ ^66^, ^67^ ^]^ Necrosis is characterized by cell rupture caused by significant physical or chemical damage and is defined as a nonprogrammed form of cell death. After cell rupture, intracellular material leaks into the extracellular space, causing the release of DAMPs that in turn are recognized by immune cells and trigger an inflammatory response. The present study found that certain intra‐ and extracellular signals cause programmed necrosis (necroptosis), and this can be significantly inhibited by caspase activity. Unlike other types of apoptosis and programmed cell necrosis, necroptosis is not dependent on caspase activity and is primarily induced by serine/threonine kinase 3 (RIPK3)‐dependent phosphorylation of mixed lineage kinase domain‐like pseudokinase (MLKL). Necroptosis is highly immunogenic and is usually induced by extracellular stimuli that cause ligands such as TNF‐*α* to bind to the death receptor (DR) in the cell membrane, ultimately causing activation downstream of RIPK.^[^ ^68^, ^69^ ^]^ Exposure of naturally high RIPK3 expressing mouse lung cancer TC‐1 cells and EL4 lymphoma cells to TNF plus Z‐VAD‐fmk and SMAC mimetic induction conditions resulted in three processes: CRT exposure, ATP secretion, and HMGB1 release during ICD induction.^[^ ^70^, ^71^ ^]^ Subsequently, controlled experiments with mouse CT26 colorectal cancer cells that do not naturally express high levels of RIPK3 confirmed that under conditions of sufficient antigenicity, ICD can induce a robust adaptive immune response through the synergistic effect of different variants.^[^ ^72^ ^]^

Regarding the mechanism of ICD, a recent study found it to be mainly induced by type I and type II inducers. Type I ICD inducers have a primary or concentrated effect in the nucleus, cytoplasm, and plasma membrane that via secondary or indirect effects induces ER stress and causes “off‐target” effects.^[^ ^73^, ^74^ ^]^ After stimulation with type I ICD inducers, including immunogenic chemotherapeutic agents and radiotherapy, PERK‐mediated activation of ER stress‐ROS signaling and eIF2a phosphorylation are followed by proapoptosis cutting of B cell receptor‐associated protein 31 (BAP31). Cis‐transporting of CRT occurs in a snap23‐dependent manner through the ER‐Golgi pathway, which exposes CRT to the plasma membrane. In addition, ER activation triggers UPR via the ATF4/CHOP pathway, which downregulates the expression of anti‐apoptotic proteins Bcl‐2 and Mcl‐1, indirectly leading to cell death.^[^ ^75^, ^76^ ^]^ An independent autophagy‐mediated pathway driven by type I ICD inducers also exists in early apoptosis, where ATP externalization is dependent on the involvement of important autophagy proteins (ATG5, ATG7, and BCN1) and other molecules.^[^ ^77^, ^78^ ^]^

Type II ICD inducers utilize ER as the main effector site, directly altering homeostasis and triggering ER stress to induce a “targeted” effect on the ICD of apoptotic cells. This pathway is dependent only on the PERK, Bax, Bak, and secretory pathways, and partially requires the involvement of caspase‐8.^[^ ^79^ ^]^ Type II inducers can induce tumor immunogenicity more efficiently than type I ICD inducers can. Hypericin‐based PDT is a typical type II ICD inducer known to induce ICD through ER stress via ROS production.^[^ ^80^, ^81^ ^]^ For ICD determination, relevant studies have also been conducted, and the corresponding detection indicators and instruments have been summarized and proposed by Fucikova et al.^[^ ^82^ ^]^

Zitvogel et al.^[^ ^83^ ^]^ defined three main ways of stimulating the immune system: increased cancer cell antigenicity, immune cell immunogenicity, and cancer cell susceptibility^[^ ^84^ ^]^ Antigenicity of tumor cells can be achieved by increasing the expression of MHCI molecules,^[^ ^85^, ^86^ ^]^ for example with oxaliplatin or cyclophosphamide, and by increasing the expression of TAA^[^ ^87^ ^]^ (for example, with fluorouracil). Some compounds promote antitumor responses by enhancing the function of immune cells (immunogenicity).^[^ ^88^ ^]^ Our group has previously conducted relevant research on the available materials for ICD induction and has made some progress. Because current photosensitizer‐based ICD inducers are not ideal for cancer immunotherapy, we developed aggregation‐induced emission materials (AIE), such as an organic photosensitizer (TPE‐DPA‐TcyP) with a distorted molecular structure, strong aggregation‐induced emission activity, and specificity, that are focused on inducing mitochondrial oxidative stress and endoplasmic reticulum stress. In contrast, other inducers, including chlorin e6 (Ce6), phospholipid A, and oxaliplatin, showed stronger immunogenicity and exerted stronger ICD induction in prophylactic tumor vaccine models.

Regarding cancer cell susceptibility, we found that ultrasound‐sensitive AIE could induce ICD and amplify antitumor activity when combined with PD‐L1.^[^ ^79^, ^89^, ^90^ ^]^ In addition, we investigated a lysosomal membrane permeabilization (LMP) inducer called TPE‐Py‐pYK (TPP)pY,^[^ ^91^ ^]^ which can induce ICD in response to alkaline phosphatase (ALP) and cause lysosomal membrane rupture, and a supramolecular self‐assembling peptide agent called DBT‐2FFGYSA)^[^ ^92^ ^]^ that not only polymerizes the EphA2 receptor but also induces ICD by inducing EphA2 overexpression in tumor cells and recruiting large numbers of tumor‐infiltrating T cells, and that promotes photothermal treatment of disease through organelle aggregation.^[^ ^93^ ^]^ We found that each of these techniques allowed for interconversion of immune cold and immune hot tumors. Additionally, near‐infrared afterglow theranostics can be used not only for surgical treatment but also to mediate tumor death by inducing sustained amplification of ICD.^[^ ^94^, ^95^ ^]^

**Figure 2:**
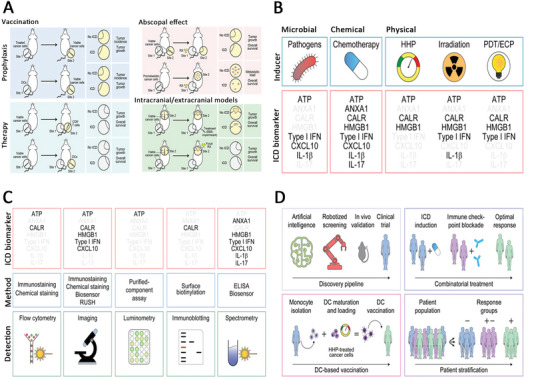
ICD detection methods and applications. A) Current methods to assess ICD in vivo, in oncological settings. Reproduced with permission.^[^ ^5^ ^]^ Copyright 2020, BMJ Publishing Group Ltd & Society for Immunotherapy of Cancer. B) Main hallmarks of ICD. C) Main methodological approaches to measure ICD biomarkers in vitro. D) Whether used alone or in combination with other drugs, the use of ICD inducers can be extended from high‐level screening to bedside care. Reproduced under the terms of the Creative Commons CC‐BY license.^[^ ^82^ ^]^ Copyright 2020, The Author(s). Pubished by Springer Nature. ICD, immunogenic cell death; HHP, high hydrostatic pressure; PDT/ECP, photodynamic therapy/extracorporeal photopheresis.

The induction of ICD having been discussed, assays for ICD can be summarized as having three main strategies (**Figure** **2**): to assess excitation of DAMPs signaling and associated stressors in dying cells, to assess the activation of APCs and their function in mediating cross‐reactivity in vivo and in vitro, and to assess the ability of dying cells to mount an adaptive immune response in an immunocompetent homologous host.^[^ ^5^ ^]^

ICD has been well described in solid tumors. Compared with these, hematological malignancies have a greater chance of contact with ICD inducers because the tumor cells grow in the bone marrow or peripheral blood at an early stage of the malignancy. After entering into the blood, an ICD inducer makes contact with these tumor cells through the circulation and is more likely to induce ICD in them than in solid tumors. Second, given that the hematological tumor cells present mostly in bone marrow or peripheral blood also circulate, but solid tumors are confined to the tumor itself, hematological malignancy cells have a greater chance of contact with immune cells in blood circulation than solid tumors do, and thus can generate a more intense anti‐tumor response. Finally, hematological malignancies have specific recognition markers with good targeting such as CD19, BCMA, and CD138. Therapies that target tumor‐specific markers, including CAR‐T and monoclonal antibodies, have shown excellent efficacy in hematological malignancies.^[^ ^96^, ^97^, ^98^, ^99^ ^]^

For these reasons, there is a great opportunity to induce powerful, targeted ICDs in hematological malignancies and provide new insights for the treatment of hematological malignancies, and strong progress in this area has been made in recent years.

## 4 The Induction of ICD in Hematological Malignancies

**Figure 3:**
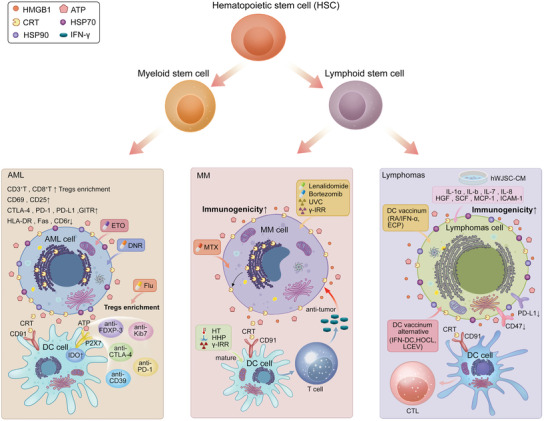
Induction of ICD in hematological malignancies. a) AML is a kind of myeloid malignancy in which large numbers of CD3 T cells and CD8 T cells can be detected in PB and enrichment of Tregs occurs. During immunopathogenesis, higher expressions of CD25 and CD69 were accompanied by higher levels of CTLA‐4, PD‐1, PD‐L1, and GITR, as well as lower levels of HLA‐DR, Fas, and CD62L. When treated with chemotherapeutic drugs, ATP release from AML cells causes IDO1 upregulation of DC via the P2×7 receptor. Furthermore, induction chemotherapy in AML patients leads to an enrichment and activation of most inhibitory Treg subsets expressing FOXP‐3, CTLA‐4, CD39, PD‐1, and Ki‐67, ultimately, establishing an immunosuppressive microenvironment. Among the chemotherapy treatments, Eto was comparable to DNR in inducing ICD events, but Flu was significantly more effective in inducing Tregs. b) MM is a plasma cell malignancy. Lenalidomide, bortezomib, and UVC, can all improve the immunogenicity of dead cancer cells used to load DCs, improve the ability of DCs to stimulate effector cells, and enhance the anticancer T cell response in vivo. MM cells treated with mitoxantrone (MTX) can develop an immunogenic cascade for ICD induction of MM by inducing CRT exposure. Moreover, different ICD‐associated induction patterns, such as *γ* ‐IRR, HHP, and HT, can upregulate the maturation markers of DCs and produce antigen‐specific T cell responses both in vivo and in vitro. c) Lymphomas is a heterogeneous group of lymphoid malignancies. In lymphoma cells loaded with DC vaccines (e.g., RA/IFN *α*, ECP, etc.) or DC vaccine replacement therapy (e.g., IFN‐DC, HOCl, LCEV), DCs show enhanced functional maturation and activation; further, enhanced maturation and activation of CTLs that more effectively recognize and specifically kill lymphoma cells, increases the efficiency of cell phagocytosis. Furthermore, treatment of lymphoma cells with human Wharton's jelly stem cell‐conditioned medium (hWJSC‐CM) enriched with medium containing IL‐1a, IL‐6, IL‐7, IL‐8, HGF, SCF, MCP‐1, and ICAM‐1 could downregulate the expression of PD‐L1 and CD47 and, in turn, enhance the antitumor effect. ICD, immunogenic cell death; AML, acute myeloid lymphoma; PB, peripheral blood; Tregs, Regulatory cells; CTLA‐4, Cytotoxic T‐lymphocyte antigen‐4; PD‐1, Programmed death‐1; PD‐L1, Programmed death‐ligand 1; GITR, glucocorticoid‐induced tumor necrosis factor receptor; DC, dendritic cell; Eto, etoposide; DNR, daunorubicin; Flu, fludarabine; UVC, ultraviolet C radiation; MM, multiple myeloma; CRT, calreticulin; *γ*‐IRR, *γ*‐irradiation; HHP, high hydrostatic pressure; HT, hyperthermia; RA, 9‐cis‐retinoic acid; ECP, extracorporeal photochemotherapy; HOCL, hypochlorite acid; LECV, lymphoma cell‐derived extracellular vesicles; CTLs, cytotoxic T cells.

Hematopoiesis is a dynamic process that maintains the stability of blood cells through self‐renewal and differentiation of hematopoietic stem cells (HSCs). The process of hematopoietic stem cell self‐renewal and pluripotent differentiation is regulated by internal and external factors, and any interruption or error in this process can lead directly to life‐threatening blood disorders.^[^ ^100^, ^101^ ^]^ Hematological malignancies can occur at any stage of blood cell development and result in abnormalities in the quantity and/or quality of blood cells, which may lead to failure in fighting infection or uncontrolled bleeding. Hematopoietic stem cells in the bone marrow (BM) give rise to immature progenitor cells of the myeloid or lymphoid lineage. Erythrocytes, platelets, and leukocytes such as neutrophils, dendritic cells, eosinophils, and macrophages form the myeloid lineage, whereas the lymphatic lineage includes the B and T lymphocytes involved in the adaptive immune response. Abnormalities in normal hematopoietic differentiation can lead to three major types of blood cancer: leukemia, lymphoma, and myeloma (**Figure** **3**, **Table** **1**). In addition to tumor cell characteristics, the suppression of immune system function is obvious in hematological malignancies,^[^ ^102^ ^]^ resulting in the formation of a suppressive immune microenvironment, a phenomenon that manifests mainly in the decreased capacity of the immune cells that exert a killing function (e.g., NK and CTL) and the increased capacity of cells that exert a suppressive function (e.g., T_regs_). As mentioned above, chemotherapy, physical cues, and materials can produce DAMPs and induce the appearance of ICD, with these in turn activating DCs, generating immune responses for antitumor activity, and restoring damaged immune cell functions; thus, ICD is a potential therapy for reactivating immune function in hematological malignancies.

**Table 1:** Current ICD inducers in hematological malignancies

	Refs.	Compound	Compound class	ICD‐hallmark	In vivo/vitro model used as evidence for antitumor immunity
Multiple Myeloma (MM)	[119]	Bortezomib	Proteasome inhibitor	HSP90	U266
	[120]	Bortezomib	Proteasome inhibitor	Ecto‐calreticulin	5T33vt
		+Melphalan	Alkylating agent	Ecto‐calreticulin Type I IFN	5T33vt
		+Decitabine+quisinostat	Epigenetic‐modulating compound	Type I IFN	5T33vt
		+Mitoxantrone	Type II topoisomerase inhibitor	Ecto‐calreticulin	5T33vt
	[122]	Belantamab mafodotin	a BCMA‐targeting antibody‐drug		NCI‐H929 EL4‐Luc‐2‐hBCMA
	[124]	MHA	a *ω*‐3 polyunsaturated fatty acid found in fish oil	Ecto‐calreticulin HSP90 HMGB1	RPMI‐8226 OPM‐2
	[125]	SIX2G	Microtubule‐targeting drug (MTA)	Ecto‐calreticulin	NCI‐H929,MM.1S, JJN‐3
Lymphomas	[131]	RA/IFN*α*	DC‐based vaccination	Ecto‐calreticulin ATP HSP70/HSP90 HMGB1 Type I IFN	Mino, SP53 and Granta 519 MCL cell lines, DOHH2 DLBCL cell line
	[133, 136]	hWJSC‐CM	Conditioned medium	Activated and mature DC cells	Ramos, CRL 1596, ATCC
Acute myeloid leukemia (AML)	[145]			Ecto‐calreticulin	C1498 C57BL/6 mice
	[147]	Cytarabine, daunorubicin, mitomycin	Cytotoxic drugs	Ecto‐calreticulin ATP HSP70/HSP90 HMGB1	Primary human AML cells
	[148]	Doxorubicin, Dororubicin	Anthracycline chemotherapeutic agents	Ecto‐calreticulin Angiostatin	HL‐60, Kasumi‐1, and primary human AML cells
	[149]	Etoposide	Antineoplastic drugs	Ecto‐calreticulin ATP HMGB1	HL‐60, KG‐1, and primary AML cell
		Daunorubicin	Anthracycline chemotherapeutic agents	Ecto‐calreticulin ATP HMGB1	HL‐60, KG‐1, and primary AML cell

### 4.1 Multiple Myeloma

MM is a malignant clonal proliferative disease of plasma cells and the second most common hematological malignancy in adults. It accounts for 10–20% of hematological tumors, with an annual incidence of 4.5–6 cases per 100 000 people, and cannot yet be cured.^[^ ^103^, ^104^ ^]^ Previous studies have shown that the BM microenvironment plays an important role in the development of MM and proliferation of malignant plasma cells.^[^ ^105^ ^]^ MM is a typical immunodeficiency disease that can be detected in both the T and B cells. MM antibody production and resulting immunoglobulin levels produce significant defects in reduced BM B cell progenitor cells,^[^ ^106^ ^]^ whereas general destruction of the T‐cell immune spectrum leads to an abnormal CD4^+^/CD8^+^ ratio.^[^ ^107^, ^108^ ^]^ Meanwhile, MM highly expresses PD‐L1 (an immune checkpoint inhibitory ligand), which contributes to tumor cell immune escape.^[^ ^109^ ^]^ In addition, BM stromal cells (MDSCs), intercell–cell exosome‐mediated secretion of cytokines, and impaired function and phenotypic changes in DCs play important roles in the pathogenesis of MM. In recent years, immunotherapy programs for MM have effectively extended patient survival time, with overall survival (OS) extended by 6–10 years from the age of diagnosis.^[^ ^110^, ^111^ ^]^ However, this is accompanied by the development of relapse or drug resistance in most patients and the progressive shortening of the effective period of each treatment.^[^ ^112^ ^]^ New drugs that have emerged in recent years (e.g., second‐generation proteasome inhibitors, third‐generation immunomodulatory drugs, histone deacetylase inhibitors, immune checkpoint inhibitors (ICIs), and monoclonal antibodies have led to improved survival in patients with MM; however, it has been difficult to improve the prognosis of patients at high risk of relapse or refractory disease.^[^ ^113^ ^]^ These immunotherapies may benefit patients with MM by inducing ICD, achieving an effective immune response, or creating an improved tumor microenvironment.^[^ ^114^ ^]^

**Figure 4:**
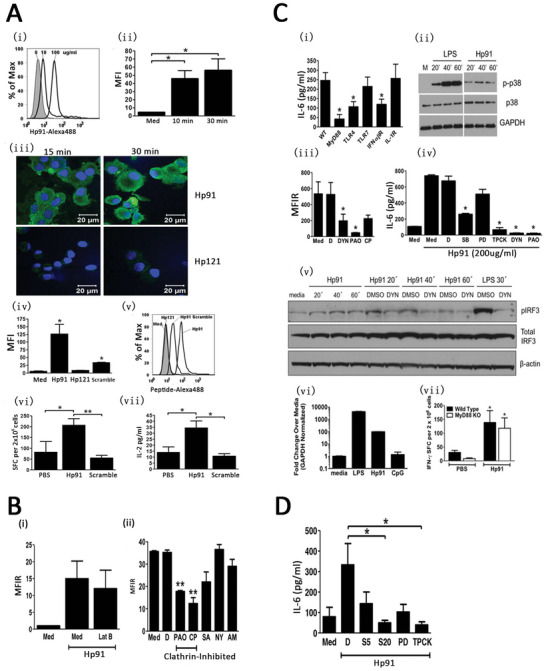
TLR4‐dependent activation of dendritic cells by an HMGB1‐derived peptide adjuvant. A) Hp91 uptake by dendritic cells is dose‐, time‐, and sequence‐dependent. B) Uptake of Hp91 is conducted via clathrin‐mediated endocytosis. C) TLR4, MyD88, and MyD88‐dependent and ‐independent pathways are necessary for Hp91‐mediated activation of antigen‐presenting cells. D) p38MAPK and NF‐*κ*B signaling are necessary for Hp91‐mediated IL‐6 secretion by dendritic cells. Reproduced under the terms of the Creative Commons CC‐BY license.^[^ ^27^ ^]^ Copyright 2014, The authors. Licensee BioMed Central Ltd.

In MM, these tumor cells are critically dependent on the presence of the UPR growth arm, which is why they are extremely sensitive to ICD induction, in which DCs play an important role.^[^ ^115^ ^]^ In healthy individuals, DCs are important for both innate and adaptive immunity (**Figure** **4**). Due to the imbalance of the BM microenvironment of MM cells and impaired function of the DC population, the myeloma cell antigen cannot present a signal to DCs because of the lack of costimulatory signals such as CD80 and CD86, which leads to defects in the antitumor response. Related studies have shown that ICD inducers (e.g., irradiation, HT, HHP, bortezomib, and lenalidomide) can significantly increase the immunogenicity of dead cancer cells, enhance the action of DCs in vivo, and ultimately enhance anticancer T‐cell responses.^[^ ^116^, ^117^, ^118^ ^]^ Bortezomib has been shown to be an effective ICD inducer and through related studies has also been shown to play a role in the induction of ICD in myeloma cells. A 2007 study induced ICD in MM cells with the proteasome inhibitor bortezomib and measured the tumor‐response rates of IFN‐*γ*‐producing T cells induced by tumor‐burden DCs. The results suggest that bortezomib‐induced myeloma cell death is more capable of activating DCs than common myeloma therapies like dexamethasone and irradiation. A possible reason for this difference is that it provides unique immune activation stimuli. The immunogenic effect of these bortezomib‐killed tumor cells depends on cell‐cell contact, which may be related to the expression of HSP90,^[^ ^50^ ^]^ a conclusion that was verified by Moeller et al.^[^ ^119^ ^]^

**Figure 5:**
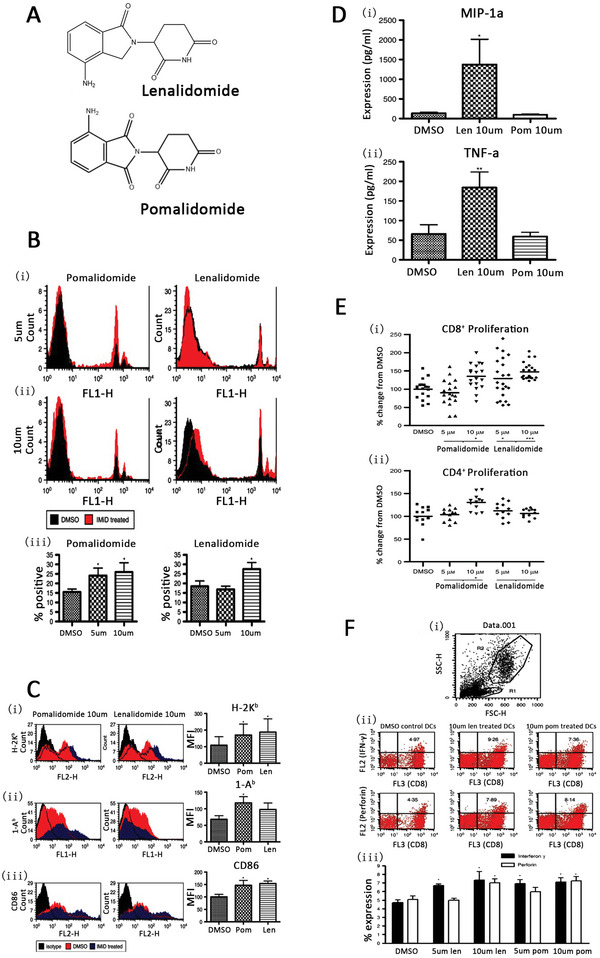
Enhanced cross‐priming of naive CD8^+^ T cells by dendritic cells treated with the IMiD immunomodulatory compounds lenalidomide and pomalidomide. A. Molecular structure type of lenalidomide and pomalidomide. B) Lenalidomide and pomalidomide increase fluorescent bead uptake. IMiD‐treated DCs that were treated with fluorescent latex beads endocytosed significantly more beads than untreated DCs (ii,iii) when analyzed by flow cytometry (i). C) Lenalidomide and pomalidomide alter phenotypic marker expression on DCs. D) Lenalidomide effects DC cytokine expression. E) Lenalidomide and pomalidomide increase DC‐dependent T‐cell expansion. F) CD8 (OTI) T cells expanded by pomalidomide‐ and lenalidomide‐treated DCs are more active than CD8 (OTI) T cells cultured with DMSO‐pre‐treated DCs. Reproduced with permission.^[^ ^123^ ^]^ Copyright 2013, John Wiley & Sons Ltd. DCs, dendritic cells; DMSO, dimethyl sulfoxide.

**Figure 6:**
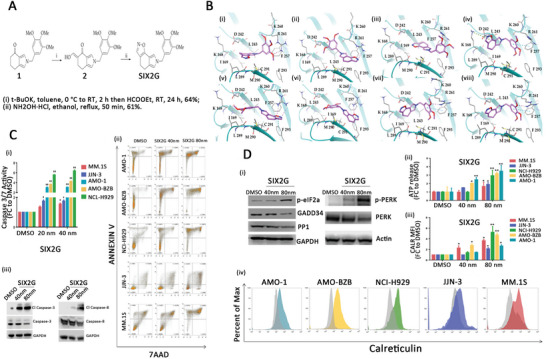
MTX can induce the production of ICD in MM cells. A) A new molecular structure of SIX2G for MTX. B) 3D representation of the best‐docked poses of SIX2G with the RVxF domain. C) Apoptosis Assays. D) Assessment of the onset of ICD. The release of ATP in the cell supernatant was detected by a Cell Titer Glo Assay. CRT exposure to the cell membrane was evaluated via flow cytometry. Reproduced under the terms of the Creative Commons CC‐BY license.^[^ ^125^ ^]^ 2022, The Authors. Published by MDPI. MTX, mitoxantrone; ICD, immunogenic cell death; CRT, calreticulin.

In light of the finding that bortezomib induces ICD in myeloma cells and enhances the function of DCs, many studies have investigated the mechanism of action of bortezomib‐induced ICD. De Beck et al.^[^ ^120^ ^]^ developed a syngeneic immunoactive 5T33MM model for a vaccine consisting of 5T33vt cells using bortezomib, melphalan, DNA methyltransferase inhibitor (decitabine), histone deacetylase inhibitor (quincitabine), or decitabine and found that the main mechanism of ICD induction is the translocation of CRT and type I interferon release. In 2021, an experiment on ICD induction with bortezomib in MM demonstrated that ICD with CRT exposure and viral simulation is an important clinical feature of bortezomib in MM therapy and indicated that another effective anti‐MM drug, belantamab mafodotin (August 2020, FDA approval for relapse or refractory), could also act as an ICD inducer.^[^ ^121^, ^122^ ^]^ In addition to bortezomib, the immunomodulators lenalidomide and pomalidomide have been identified as common inducers of ICD in myeloma cells. Lenalidomide and pomalidomide, as drugs with anti‐inflammatory, immunomodulatory, or anticancer activity, have been shown to enhance the cross‐initiation of naive CD8^+^ T cells (up to 47%, both lenalidomide and pomalidomide) and CD4^+^ T cells (30%, pomalidomide alone), Thus, these drugs enhance the uptake of DCs, increasing the efficacy of antigen presentation and supporting their use in DC vaccine therapy (**Figure** **5**).^[^ ^123^ ^]^ Apart from chemotherapy drugs, D'Eliseo et al. found that the immunogenicity of cell death induced by docosahexaenoic acid (MHA, an *ω*‐3 polyunsaturated fatty acid found in fish oil) in MM cells was not only related to cell surface exposure of CRT, but also to HSP90 release and the extracellular release of HMGB1.^[^ ^124^ ^]^ And in 2022, Grillone et al. found that MM cells treated with the microtubule‐targeting drug (MTA) SIX2G could induce CRT exposure through interaction with the PP1 RVxF domain followed by an immunogenic cascade, suggesting that this treatment could also be used for ICD induction of MM (**Figure** **6**).^[^ ^125^ ^]^ In addition, Hyp‐PDT can induce the complete regression of myeloma cells under both therapeutic and prophylactic vaccination conditions in vivo.^[^ ^126^ ^]^ In our previous work, we explored a novel strategy for enhancing ICD immunotherapy in myeloma cells in support of MM treatment: the use of aggregation‐induced emission (AIE)‐loaded bovine serum albumin (BSA) nanoparticles (called BSA/TPA‐Erdn) to increase production of reactive oxygen species in mitochondria, which induces ICD in myeloma cells, activates T cells, and reverses the senescence of T cells, restoring the immune microenvironment.^[^ ^127^ ^]^

The current clinical use of chemotherapeutic drugs (e.g., bortezomib, belantamab, mafodotin, potomalidomide, and lenalidomide) can promote DC function, and novel drugs (e.g., MTA) can induce ICD production by enhancing exposure of CRT, which then plays a role in enhancing the immune response. Determining whether ICD induction in MM occurs through other pathways or mechanisms of action requires further exploration; however, discovery of other effective ICD inducers, drug combinations that stimulate stronger ICD induction, or even specific targeting of myeloma cells with ICD therapy should be expected.

### 4.2 Lymphomas

A lymphoma is a heterogeneous lymphoid malignancy. Therapeutic chemotherapy for classical Hodgkin's lymphoma (CHL) ranges from a typical CHL regimen using ABVD (containing doxorubicin (DOX [Adriamycin], bleomycin, vinblastine, and dacarbazine) to a CHOP‐like chemotherapy regimen supplemented with rituximab; however, a standardized treatment model has not yet been established.^[^ ^128^ ^]^ Currently, two thirds of NHL patients can achieve long‐term control or cure, but treatment options are limited for relapsed and refractory patients. Regarding T‐cell lymphomas, CHOP (cyclophosphamide, DOX, vincristine, and prednisolone) or CHOP‐like chemotherapy remains the standard approach for most peripheral T‐cell lymphomas. However, most patients experience poor treatment outcomes.

The BM microenvironment of lymphomas varies among lymphoma types. Some tumor microenvironments such as those observed in follicular lymphoma are dominated by T cells, whereas in other tumor microenvironments, lymphocytes predominate over macrophages, as in the case of the B cells and macrophages observed in Burkitt's lymphoma.^[^ ^129^ ^]^ Although the mechanism of the BM microenvironment in various types of lymphoma is still unclear, immunotherapy aimed at improving tumor‐specific T‐cell responses, eradicating residual malignant cells, and preventing disease recurrence has achieved efficacy. Furthermore, improved efficacy of combination therapy is expected in the future.^[^ ^130^ ^]^ Since tumor cells receiving ICD can show superior immunogenicity and promote a strong antitumor response mainly biased toward Th1 immunity, much progress has also been made in the context of lymphoma cells.

**Figure 7:**
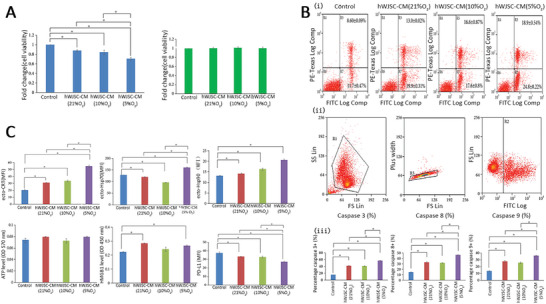
hWJSC‐CM can enhance the activity of DCs and the immune response against lymphoma cells. A) Cell viability (MTS assay) of human lymphoma and control CCD112sk cells exposed to normoxic and hypoxic hWJSC‐CM for 48 h. Bi) Apoptotic assay (dot plots) of lymphoma cells after treatment with normoxic and hypoxic hWJSC‐CM. (ii‐iii). Flow cytometry dot plots and percentage caspase activity showed significant increases in caspase 3, caspase 8, and caspase 9 activities in lymphoma cells after treatment with hWJSC‐CM compared to the control. C) Immunogenic cell death analysis and the secreted ATP and HMGB1 levels of lymphoma cells after treatment with normoxic and hypoxic hWJSC‐CM. Reproduced under the terms of the Creative Commons CC‐BY license.^[^ ^136^ ^]^ Copyright 2020, The Authors. Published by Hindawi. hWJSC‐CM, human Wharton's jelly stem cell‐conditioned medium; DCs, dendritic cells; HMGB1, high mobility group box 1.

Currently, DC‐based vaccines have a promising future in the treatment of lymphoma; however, the clinical therapeutic benefit is limited, and selection of the optimal antigenic agent has become an emerging issue. Many studies have begun to consider new strategies to improve DC vaccines for lymphoma by using ICD to enhance the immune response. Treatment of diffuse large B‐cell lymphoma (DLBCL) and mantle cell lymphoma (MCL) cell lines with 9‐cis‐retinoic acid (RA) and IFN‐*α* was found to exert therapeutic effects through the induction of CRT outgrowth, early HSP70/90 membrane exposure, CD47 downregulation, and enhanced HMGB1 secretion. Meanwhile, experiments showed that DCs loaded with RA/IFN*α*‐TCLs exhibited enhanced phagocytosis when highly immunogenic tumor cell lysates (TCLs) and apoptotic cells were obtained from lymphoma cells treated with RA/IFN*α* and undergoing ICD.^[^ ^131^ ^]^ Meanwhile, researchers have developed alternatives to DC‐based vaccines; lymphoma cell‐rich IFN‐DC acts as a powerful inducer of a specific anti‐lymphoma immune response to induce ICD; hypochlorite acid (HOCl) is a strong fungicide that enhances protein immunogenicity and may be applicable to many cancer types, including lymphoma; and lymphoma cell‐derived extracellular vesicles (LCEV) can also potentially load DC as an alternative antigen.^[^ ^132^ ^]^ These new treatment strategies offer benefits to patients with relapsed or refractory lymphoma. In 2017, Lin et al.^[^ ^133^ ^]^ showed that ICD induction of lymphoma cells could be performed through exposure to medium conditioned with human umbilical cord jelly (hWJSC‐CM), which provided activation and stimulation of mature DC to dying lymphoma cells, thereby inducing tumor‐specific T‐cell responses. The 3 kDa MWCO concentrate of hWJSC‐CM inhibits the expression of defense molecules in lymphoma cells by inducing production of DAMPS, which in turn makes the lymphoma cells more susceptible to attack by the host immune cells and increases the chances of complete remission. Meanwhile, downregulation of PD‐L1 and CD47 was observed after exposure to the hWJSC‐CM concentrate, which implies a significant enhancement of the antitumor effect.^[^ ^134^ ^]^ Proteomic analysis of hWJSC‐CMs revealed increased expression of factors associated with the regulation of cancer cell death and the immune system, suggesting that these proteins play a role in inducing lymphoma cells to kill tumor cells and modulate the immune response.^[^ ^135^ ^]^ In 2020, the authors further found that hypoxic hWJSC‐CM had greater tumor‐killing ability than normoxic hWJSC‐CM; therefore, new drugs against malignancy using hypoxic hWJSC‐CM should be preferred (**Figure** **7**).^[^ ^136^ ^]^

The current method for ICD induction of lymphoma involves two modes: using ICD strategies to enhance the immune activity of DC vaccines, and using the corresponding culture medium for DC stimulation. Other studies on the induction of ICD in lymphoma cells to improve immunogenicity of apoptotic cells are ongoing, aimed at providing new diagnostic and treatment options and prolonging overall survival in patients with relapsed or refractory lymphoma.

### 4.3 Myeloid Malignancies

The list of characteristic myeloid malignancies includes myelodysplastic syndromes (MDS), chronic myeloproliferative neoplasms (MPN), and acute myeloid leukemia (AML).^[^ ^137^, ^138^ ^]^ Peripheral blood from patients with AML can show an increase in CD3^+^ and CD8^+^ T cells and enrichment of T_regs_ compared to blood from healthy donors. The relatively high expression of immune markers such as CD25 and CD69 and increased levels of the regulatory molecules cytotoxic T lymphocyte‐associated protein 4 (CTLA‐4), PD‐1, and PD‐L1, as well as lower levels of HLA‐DR, Fas, and CD62L, suggest that the immune system of AML patients is in a comparative state of initiation or activation.^[^ ^139^, ^140^, ^141^ ^]^ Relevant studies have confirmed the presence of immune imbalance and escape in both AML and MDS patients. Elevated MDSC levels in MDS produce excess ROS and TGF‐*β*, exerting a suppressive effect on T cells.^[^ ^142^ ^]^ The mechanism of immune escape in MPN patients is associated with reduced expression of genes related to antigen processing and presentation and can be accompanied by a marked disturbance in the cytokine environment and some degree of functional deficiency in their T cell function.^[^ ^143^, ^144^ ^]^ All in all, immune dysfunction increases myeloid malignancy, which paves the way for use of ICD therapy in hematological malignancies.

**Figure 8:**
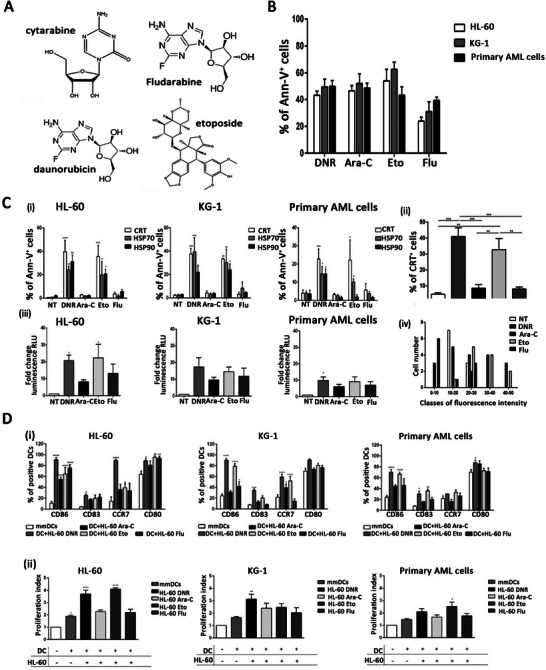
Differences in immunogenicity of chemotherapy in acute myeloid leukemia. A) The molecular formula of (Ara‐C), (DNR), Eto, and Flu. B) Flow cytometry analysis of AML cell apoptosis after chemotherapy treatment. C‐i) Flow cytometry analysis of CRT and HSP translocation on the cell‐surface of AML cells after chemotherapy treatment. (ii‐iv). Immunofluorescence analysis of CRT translocation on the cell‐surface of HL‐60 cell lines, ATP release from HL‐60, KG‐1, primary AML cells, and HMGB1 release from the nucleus after chemotherapy treatment. D‐i) Flow cytometry analysis of DC maturation mediated by HL‐60, KG‐1, and primary AML cells treated with chemotherapy. (ii). Flow cytometry analysis of CD3+ T cell proliferation mediated by DCs loaded with chemotherapy‐treated HL‐60, KG‐1, and primary AML cells. Reproduced under the terms of the Creative Commons CC‐BY license.^[^ ^149^ ^]^ Copyright 2020, The Authors. Published by MDPI. Eto, etoposide; Flu, fludarabine; AML, acute myeloid leukemia; CRT, calreticulin; HSP, heat‐shock protein; DC, dendritic cell.

Plasma membrane transport of CRT and resulting activation of the UPR is a key step in the induction of ICD. Chen et al. found that CRT exposure induced apoptosis of tumor cells and promoted tumor antigen presentation to T cells in an in vitro mouse AML model; the authors also found that CRT directly stimulated APCs, increased tumor‐specific T cells, and promoted the induction of effective leukemia‐specific T cell immunity associated with the host IFN‐I response.^[^ ^145^ ^]^ Chemotherapeutic agents (e.g., anthracyclines) as ICD inducers have been shown to activate the immune response through DC‐based antitumor T lymphocyte cross‐initiation in solid tumors and hematological malignancies. In in vivo AML mouse models, anthracycline chemotherapeutic agents were observed to trigger ICD and transfer CRT from the nucleus to the surface of leukemia cells; cultured AML cells in vitro showed spontaneous release of HSP70/90.^[^ ^146^, ^147^ ^]^ A study on CRT regulation in AML serum after anthracycline treatment found that this antibiotic causes release of an elastase‐inert N‐terminal CRT peptide into the serum. This CRT peptide was identified as angiostatin, a peptide that blocks the differentiation of ATRA‐triggered leukemic cells. Since the serum angiostatin level is inversely correlated with BM vascularization in patients with AML, it can exert anti‐angiogenic effects and can be used in clinical treatment.^[^ ^148^ ^]^ A recent study showed that daunorubicin (DNR) plays a stronger role in inducing ICD than does azacitidine (Ara‐C). A 2020 study^[^ ^149^ ^]^ treated AML cells with DNR, Ara‐C, etoposide (Eto), and fludarabine (Flu) and compared CRT and HSP70/90 translocation, HMGB1, and ATP release (**Figure** **8**). Like Ara‐C, Flu was unable to induce ICD, but Flu had a significant effect on the induction of T_regs_. Alternatively, Eto‐induced ICD is similar to DNR.

However, during ICD after chemotherapy, AML cells can release ATP, upregulating IDO1 on DCs via the P2×7 receptor and enriching T_regs_, which establishes an immunosuppressive microenvironment. ATP released from chemotherapy‐treated dying leukemia cells functions during ICD to induce an immunosuppressive microenvironment through T_regs_ and IDO1‐expressing DCs; these findings reflect the enrichment and activation of suppressive T_reg_ subpopulations (including CTLA‐4, FOXP‐3, PD‐1, CD39, and Ki‐67) that can occur in T cells of AML patients after chemotherapy induction.^[^ ^2^, ^150^ ^]^ This study suggests the possibility of combining anti‐PD‐1 checkpoint inhibitors with chemotherapy to target T_regs_, given that T_regs_ obtained after co‐culture with DCs using AML cells treated with Flu had the highest PD‐1 expression.

ICD induction in myeloid tumors is mainly conducted via treatment with an anthracycline chemotherapeutic drug, which exerts antitumor effects by mediating ICD production. However, few clinical studies are reported for other myeloid tumors like MDS and MPN, and further research on efficient ICD inducers is needed.

### 4.4 Lymphocytic Leukemia

Lymphocytic leukemia includes acute lymphoblastic leukemia (ALL) and chronic lymphocytic leukemia (CLL),^[^ ^151^ ^]^ which are characterized by the pathological proliferation of lymphocytes. In recent years, the following targeted BCR therapies have been proposed: ABL1 tyrosine kinase inhibitors, CD19 directional CAR‐T cell therapy, an antibody‐drug combination (enozumab‐ozomi), a monoclonal antibody (rituximab), and a dual‐specificity antibody (blinatumomab). Some new therapies can initially achieve remission, but their efficacy is limited for relapsed and refractory patients.^[^ ^152^, ^153^ ^]^ ICD induction has a corresponding progression in such diseases. The CD47 agonist peptide PKHB1, hyaluronic acid nanoparticles, *Withania somnifera*, and photodynamic therapy can all be used to induce ICD in lymphocytic leukemia cells.^[^ ^154^, ^155^, ^156^, ^157^ ^]^ But, more efficient ICD inducers need to be explored.

Reports on ICD induction in other rare hematological malignancies such as POEMS syndrome, hemophagocytic syndrome, and special types of non‐Hodgkin's lymphoma are even scarcer, and further exploration is needed to improve this area and thus bring benefits to patients with these rare diseases.

Although many results from in vitro and in vivo experiments support use of ICD induction in hematological malignancies to effectively enhance the immune response and exert antitumor activity, data from clinical trials are lacking. In our view, the reasons why ICDs have not yet become widely available in clinical studies include the following. The main reason is that there are few clear and efficient inducers of ICDs in hematological malignancies. Although some chemotherapeutic drugs, physical cues, and other materials have been found to induce ICDs, the intensity of the intracellular response is variable; therefore, even under the same stimulation of DAMPs, the same inducer can produce different efficacies in different cells, or even fail to induce ICD. Second, the issue of targeting has not yet been properly addressed. Further studies are needed to target ICD inducers in specific cell types such as tumor cells. Some tumor‐specific markers like BCMA, CD19, and GPRC5D have been used as CAR‐T therapy markers for MM, and CD19 has been used for B‐cell malignancies. It is well known that the key aspects of ICD induction are ER stress and mitochondrial ROS generation, and it is also possible to target these specific organelles (i.e., the endoplasmic reticulum and mitochondria) for ICD induction.

Given that efficient ICD inducers are currently scarce and those that are mostly used in hematological malignancies are highly toxic chemotherapeutic agents, some newly reported materials that can induce ER stress‐based ICD are being extensively studied. One study used heptamethine cyanine, which is easily enriched at tumor sites, as a reagent for targeting and imaging tumor genes. This enhanced molecular targeting of the endoplasmic reticulum, which in turn enhanced the efficacy of molecular photodynamic therapy and photothermal therapy, and finally induced ICD through ER stress to produce antitumor effects.^[^ ^158^ ^]^ In addition, a new ICD induction paradigm based on mitochondria‐provoked stress magnetic heat therapy (MHT) was discovered and found to be effective in stimulating tumor‐associated macrophages (TAMs) to exert antitumor effects.^[^ ^159^ ^]^ These related research advances provide new ideas for clinical translation. However, the effects of ICD inducers in hematological malignancies require further study.

Considering that these issues remain unresolved, current ICD induction‐related immunotherapeutic strategies, although promising, have not yet been implemented in clinical trials or in patients. Moreover, how to combine the currently known modalities capable of inducing ICDs with clinically used therapies to improve the treatment effect in hematological malignancies is also a question that needs to be considered.

## 5 Combined Use of ICD Inducers and Other Therapies for Hematological Malignancies

**Figure 9:**
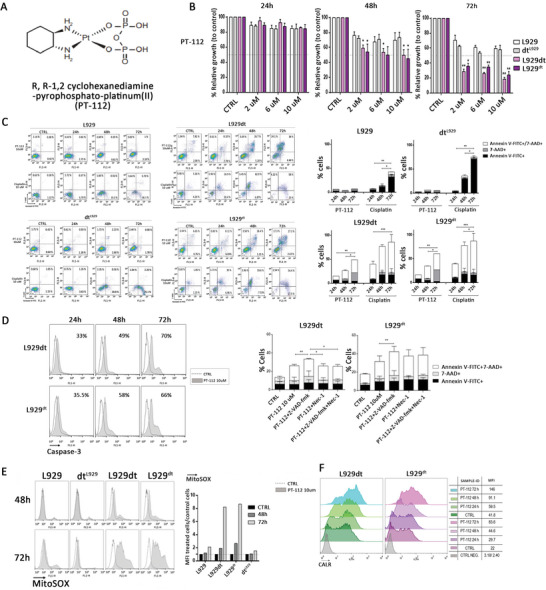
PT‐112 induces mitochondrial stress and immunogenic cell death, targeting tumor cells with mitochondrial deficiencies. A) The molecular formula of PT‐112. B) Cell growth analysis after treatment with PT‐112. C) Cytotoxicity of PT‐112. Parental L929, L929dt and cybrids cells were incubated with 10 µM of PT‐112 or cisplatin for 24, 48, and 72 h. Then, cells were simultaneously stained with annexin‐V‐FITC and 7‐AAD and analyzed by flow cytometry. D) Analysis of mitochondrial membrane potential (∆Ψm) upon treatment with PT‐112 across different incubation times. E) Analysis of total and specific mitochondrial ROS production upon treatment with PT‐112 across different incubation times. F) CRT exposure upon PT‐112 treatment. Reproduced under the terms of the Creative Commons CC‐BY license.^[^ ^168^ ^]^ Copyright 2022, The Authors. Published by MDPI. ROS, reactive oxygen species; CRT, calreticulin.

The immune microenvironment is a key driver and regulator of leukemia progression and hematological malignancy. Dysregulation of immune cell status is a hallmark of hematological malignancy and tumor formation; this malignant immune adaptation has profound effects on leukemic primitive cell proliferation, disease spread, and drug resistance.^[^ ^160^ ^]^ Most hematological malignancies have an immune imbalance; therefore, adjusting the status of the immune microenvironment is important for the improvement of patient prognosis. We infer that therapies for hematological tumors that are based on ICD have improved prospects for clinical efficacy and development. A recent study found that some materials or chemical elements that can be used to regulate the immune environment are being used for the treatment of disease.^[^ ^161^, ^162^ ^]^ Therefore, there is rising interest in combining induced ICD immunotherapy with other therapies to treat hematological malignancies. Consequently, in recent years, many studies have considered adding ICD inducers to immunotherapy to achieve better clinical efficacy (**Figure** **9**).

**Figure 10:**
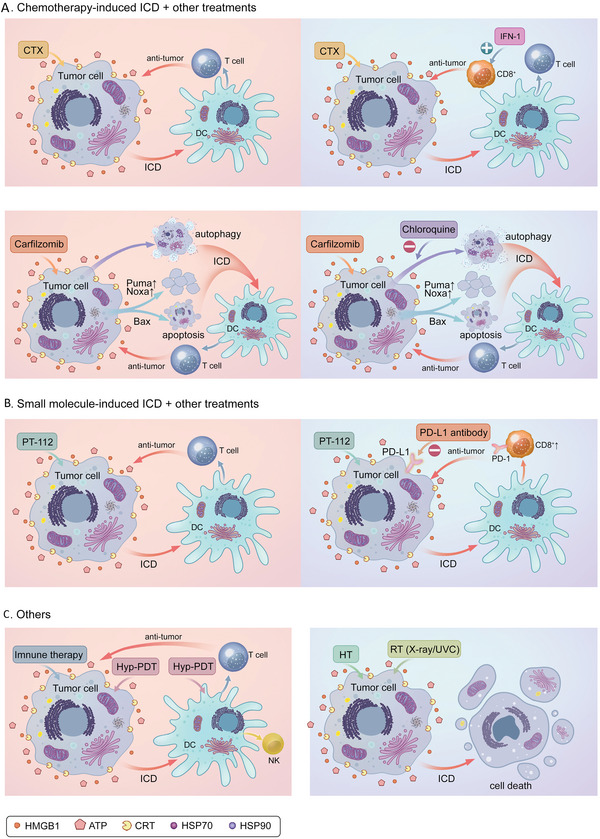
Combination of ICD inducers with other therapies for hematological malignancy treatment. A) Chemotherapy‐induced ICD and other treatments. CTX can directly induce the ICD of tumor cells, and then promote DC activation and maturation, stimulate the generation of T cells, and exert their antitumor effects. In combination with IFN‐1, IFN‐1 promotes DC‐mediated CD8 T cell responses and exerts synergistic therapeutic antitumor effects with CTX in vivo. Carfilzomib can induce cell apoptosis by both Puma and Noxa upregulation, interaction with Bax, and induction of autophagy. Through the danger signals emitted by apoptotic cells, carfilzomib can increase CRT exposure, induce ICD of tumor cells, then produce an immune response. Chloroquine can inhibit the carfilzomib‐mediated autophagy processes, enhance the cellular apoptotic processes both in vitro and in vivo, and enhance the antitumor effects. B) Small molecule‐induced ICD and other treatments. The cytotoxic effects of PT‐112 are closely related to NAMPs signaling, including the exposure to CRT and the secretion of ATP and HMGB1, which drive ICD and exert antitumor immune effects. When combined with an anti‐PD‐L1 mAb, PT‐112 can enhance treatment‐related antitumor immunity by increasing CD8^+^ CTL infiltration and reducing Treg cell‐dependent immunosuppression. C) Combination immunotherapy with DC loading tumor cells treated with PDT can stimulate T and NK cells to produce specific cytotoxicity. HT can act as an effective sensitizer for conventional chemotherapy or RT; the combination of HT and RT (X‐ray or UVC) can induce inflammatory necrotizing tumor death, a process that can be monitored by the release of HMGB1 and HSP70, while stimulating DC maturation and release of proinflammatory cytokines. ICD, immunogenic cell death; CTX, cyclophosphamide; DC, dendritic cell; IFN‐1, Interferon‐1; Hyp‐PDT, hypericin‐based photodynamic therapy; NAMPs, nematode‐associated molecular patterns; CRT, calreticulin; OXP, oxaliplatin; HT, hypothermia; RT, radiation therapy; UVC, ultraviolet C radiation; *γ*‐IRR, *γ*‐irradiation.

1. Chemotherapy‐induced ICD with other treatments: In 2011, Schiavoni et al.^[^ ^163^ ^]^ were the first to demonstrate that cyclophosphamide (CTX) induces ICD in tumor cells. MTX acts as an ICD inducer, activating DCs by releasing large amounts of antigens, promoting DC travel toward tumor cells, and promoting the endostatic expansion of the DC pool. Given that IFN‐I can enhance the DC‐mediated humoral immune response, the possibility of combining CTX with IFN‐I to promote in vivo antitumor therapeutic effects can be considered. In 2016, Jarauta et al.^[^ ^164^ ^]^ proposed that carfilzomib, a proteasome inhibitor, could increase the proportion of cells located in the pre‐apoptotic G2/M phase, causing cells to exhibit typical apoptotic features and activate the intrinsic pathway of apoptosis. Apoptosis was induced by the upregulation of PUMA and NOXA and their interaction with Bax; carfilzomib can induce autophagy, but this process ends as apoptosis progresses.^[^ ^165^, ^166^ ^]^ When combined with chloroquine, the autophagic process of carfilzomib results in enhanced apoptosis in vitro and in vivo, increased CRT exposure via danger signals from apoptotic cells, induction of ICD in myeloma cells, and stimulation of the immune response to myeloma. Combining carfilzomib with chloroquine should be considered in the future to improve the treatment of patients with MM. Wei et al.^[^ ^167^ ^]^ proposed that an emerging strategy to enhance the efficacy of ICIs in relapsed/refractory cancer is to increase ICD by combining cytotoxic therapies, thus suggesting that ICI combined with chemotherapy may maximize the efficacy of AML treatment.

2. Small‐molecule ICD inducers and other treatments: In 2020, Yamazaki et al.^[^ ^168^, ^169^ ^]^ first demonstrated that PT‐112 acts as a novel platinum–pyrophosphate conjugate with cytotoxic effects that are similar to those of nanoparticle‐associated molecular pattern signaling, including in exposure of CRT and secretion of ATP and HMGB1, which drive ICD and exert antitumor immune effects (**Figure** **10**). By combining PT‐112 with an ICI, such as a PD‐1/PD‐L1 inhibitor, for the treatment of a mouse model, it was found that PT‐112 could reduce TAM and T_reg_ cell‐dependent immunosuppression by increasing CD8^+^ CTL infiltration and enhancing treatment‐related antitumor immunity; in particular, this had a high efficacy in immunological cold tumors. This result is consistent with clinical evidence in patients with solid tumors, where it demonstrated strong therapeutic activity either as an independent therapeutic agent (NCT02266745)^[^ ^170^, ^171^ ^]^ or in combination with PD‐L1 blockers like avelumab (NCT03409458).^[^ ^172^ ^]^ Recent studies have also found that the tyrosine kinase inhibitors crizotinib and ceritinib can induce ICD in anaplastic large‐cell lymphomas by exerting “target” or “off‐target” effects on anaplastic lymphoma kinase (ALK), either alone or in combination with other therapeutic agents.^[^ ^173^, ^174^ ^]^

3. Other options: Existing reports from mouse prophylactic and vaccination models suggest that Hyp‐PDT can mediate effective tumor rejection by triggering an ICD in the conductor. The cytotoxicity of mouse T cells and NK cells against tumors can be stimulated by photoimmunotherapy containing PDT‐treated tumor cells combined with DCs.^[^ ^175^ ^]^ Hypothermia (HT) is considered a sensitizing agent for conventional radiotherapy and chemotherapy in clinical oncology, and has been shown to improve patient overall survival in various clinical trials.^[^ ^176^ ^]^ However, HT cannot be used alone. To make HT clinically useful, an HT material based on liposomes containing magnetic nanoparticles has been developed to stimulate antitumor immune responses in vivo through necrotic death of tumor cells and the release of HSP70. Furthermore, the use of a combination of HT and radiation therapy (X‐ray or UVC) can induce inflammatory necrotizing tumor death through the release of HMGB1 and HSP70 and stimulate DC maturation and cytokine release.^[^ ^177^, ^178^, ^179^ ^]^ Many preclinical studies are currently being carried out with the hope that clinical application of Hyp‐PDT and HT‐based immunotherapy can be achieved soon. Owing to the high tumorigenic mutation rate induced by UVC light, clinical examples of UVC treatment for human tumors have not been reported.^[^ ^180^ ^]^

In conclusion, ICD inducers combined with methods like immunotherapy that enhance the antitumor immune response are promising for widespread application in treatment of hematological malignancies. However, numerous clinical experiments to confirm their efficacy and safety are still needed.

## 6 Conclusion

Over the past decade, research on ICD has increased. The key to ICD development is the launch of DAMPs, which lead to ER stress and ROS generation, resulting in a complete antigen‐specific immune response. An imbalance in the immune microenvironment of hematological malignancies is the main cause of disease progression and recurrence. Currently, immunotherapy is widely used to regulate the immune microenvironment of patients with hematological malignancies, with great clinical benefit. Here, we review ICD induction mechanisms in the hematological malignancies MM, lymphoma, myeloid malignancy, and lymphocytic leukemia with an eye toward finding more efficient and targeted ICD inducers and improving the translation of ICD‐based treatment to preclinical experiments and studies. Combined ICD inducers and immunotherapies like ICIs are promising therapies for patients with hematological malignancies. Further research is needed to clarify the safety and effects of this class of therapies.

## Conflict of Interest

The authors declare no conflict of interest.
